# Concurrently wasted and stunted children 6‐59 months in Karamoja, Uganda: prevalence and case detection

**DOI:** 10.1111/mcn.13000

**Published:** 2020-03-25

**Authors:** Gloria Adobea Odei Obeng‐Amoako, Mark Myatt, Joel Conkle, Brenda Kaijuka Muwaga, Richmond Aryeetey, Andrew Livex Okwi, Isaac Okullo, Ezekiel Mupere, Henry Wamani, André Briend, Charles Amnon Sunday Karamagi, Joan Nakayaga Kalyango

**Affiliations:** ^1^ Clinical Epidemiology Unit, School of Medicine, College of Health Sciences Makerere University Kampala Uganda; ^2^ Brixton Health Powys UK; ^3^ Health and Nutrition Section UNICEF Windhoek Namibia; ^4^ Independent Consultant Kampala Uganda; ^5^ School of Public Health University of Ghana Accra Ghana; ^6^ Department of Pathology, School of Biomedical Sciences, College of Health Sciences Makerere University Kampala Uganda; ^7^ Department of Dentistry, School of Health Sciences, College of Health Sciences Makerere University Kampala Uganda; ^8^ Department of Paediatrics and Child Health, College of Health Sciences Makerere University Kampala Uganda; ^9^ Department of Community Health and Behavioural Sciences, School of Public Health, College of Health Sciences Makerere University Kampala Uganda; ^10^ School of Medicine, Centre for Child Health Research University of Tampere Tampere Finland; ^11^ Department of Nutrition, Exercise and Sports University of Copenhagen Copenhagen Denmark; ^12^ Department of Pharmacy, College of Health Sciences Makerere University Kampala Uganda

**Keywords:** case detection, concurrent wasting and stunting, MUAC, stunting, Uganda, wasting, WaSt

## Abstract

We assessed prevalence of concurrently wasted and stunted (WaSt) and explored the overlaps between wasted, stunted, underweight and low mid‐upper arm circumference (MUAC) among children aged 6–59 months in Karamoja, Uganda. We also determined optimal weight‐for‐age (WAZ) and MUAC thresholds for detecting WaSt. We conducted secondary data analysis with 2015–2018 Food Security and Nutrition Assessment (FSNA) cross‐sectional survey datasets from Karamoja. Wasting, stunting and underweight were defined as <−2.0 z‐scores using WHO growth standards. Low MUAC was defined as <12.5 cm. We defined WaSt as concurrent wasting and stunting. Prevalence of WaSt was 4.96% (95% CI [4.64, 5.29]). WaSt was more prevalent in lean than harvest season (5.21% vs. 4.53%; *p* = .018). About half (53.92%) of WaSt children had low MUAC, and all were underweight. Younger children aged <36 months had more WaSt, particularly males. Males with WaSt had higher median MUAC than females (12.50 vs. 12.10 cm; *p* < .001). A WAZ <−2.60 threshold detected WaSt with excellent sensitivity (99.02%) and high specificity (90.71%). MUAC threshold <13.20 cm had good sensitivity (81.58%) and moderate specificity (76.15%) to detect WaSt. WaSt prevalence of 5% is a public health concern, given its high mortality risk. All children with WaSt were underweight and half had low MUAC. WAZ and MUAC could be useful tools for detecting WaSt. Prevalence monitoring and prospective studies on WAZ and MUAC cut‐offs for WaSt detection are recommended. Future consideration to integrate WAZ into therapeutic feeding programmes is recommended to detect and treat WaSt children.

**Key messages**

- WaSt prevalence of 5% raises public health concerns. In future, therapeutic feeding programmes should consider using WAZ to detect WaSt for treatment. Half (53.92%) of WaSt children had low MUAC, and all WaSt children were underweight.
- Younger children aged <36 months, particularly males, had more WaSt at 1.77 ratios. Nonetheless, majority of WaSt children who had low MUAC were females. Further analysis of the WaSt sex difference may prove useful.
- Thresholds of WAZ <−2.60 and MUAC <13.20 cm detected WaSt at optimal performance. Prospective studies on WAZ and MUAC cut‐offs for WaSt detection in different populations are recommended.

## 1 INTRODUCTION

Childhood undernutrition remains an important public health problem. Globally, stunting (height‐for‐age z‐scores [HAZ] <−2.0) and wasting (weight‐for‐ height z‐scores [WHZ] <−2.0) affects about 151 and 51 million children under 5 years, respectively (UNICEF, WHO, & World Bank Group, 2018). In Uganda, 4%, 29% and 10.5% of children under 5 years are wasted, stunted and underweight, respectively (UBOS & ICF, 2018).

Wasting is also diagnosed by mid‐upper arm circumference, that is, MUAC <12.5 cm and WHZ <−2.0 are indicators for acute malnutrition (UNHCR & WFP, 2011). Hereafter, acute malnutrition defined as MUAC <12.5 cm is referred to as low MUAC for ease of communication.

Recent evidence indicates that these different forms of undernutrition can coexist in some children (Garenne, Myatt, Khara, Dolan, & Briend, 2019; Khara, Mwangome, Ngari, & Dolan, 2018; McDonald et al., 2013; Myatt et al., 2018; Schoenbuchner et al., 2019; Wells et al., 2019). Being wasted and stunted (WaSt) has a heightened risk of death equivalent to being severely wasted as defined by WHZ <−3 (Garenne et al., 2019;McDonald et al., 2013 ; Myatt et al., 2018). WaSt children have a 12‐fold higher risk of death than children without anthropometric deficit (McDonald et al., 2013; Myatt et al., 2018). On the contrary, stunted children have 1.5‐fold risk and wasted children have 2.3‐fold risk compared with children without deficits (McDonald et al., 2013; Myatt et al., 2018; Odei Obeng‐Amoako et al., 2020). Evidence suggests that children with WaSt, similar to children with severe wasting, could benefit from existing therapeutic feeding programmes to avert mortality (Myatt et al., 2018).

A recent analysis showed a pooled WaSt prevalence of 3% with a range of 0% to 8.0% among children aged 6–59 months in 84 countries (Khara et al., 2018). Drivers of the occurrence of WaSt are yet to be fully uncovered. However, age, sex and food insecurity season have been linked to WaSt (Garenne et al., 2019; Myatt et al., 2018; Schoenbuchner et al., 2019). How to screen and detect WaSt cases at the community and health facility levels is a critical prerequisite question for future public health programmes and policy decision‐making. WAZ and MUAC are considered the appropriate anthropometric indicators for detecting WaSt cases at risk of death (Myatt et al., 2018; Myatt, Khara, Dolan, Garenne, & Briend, 2019). WAZ and MUAC for WaSt detection would potentially avoid the logistical complexities and errors associated with height measurements in the field (Myatt, Khara, & Collins, 2006).

Global and contextual evidence on prevalence and case detection of WaSt to inform decision‐making is limited. Earlier analysis on WaSt focused on concurrency of WHZ <−2.0, HAZ <−2.0 and WAZ <−2.0 (Garenne et al., 2019; Khara et al., 2018; Myatt et al., 2018; Myatt et al., 2019; Schoenbuchner et al., 2019). Our study seeks to add to the evidence on WaSt by analysing the overlaps between four conventional anthropometric deficits: WHZ <−2.0, HAZ <−2.0, WAZ <−2.0 and MUAC <12.5 cm. Additionally, this analysis provides contextual evidence on the burden and detection of WaSt in a protracted food insecure setting. We used population‐based FSNA survey datasets (June 2015–July 2018) to assess the prevalence of WaSt and explore the overlaps between WHZ <−2.0, HAZ <−2.0, WAZ <−2.0 and MUAC <12.5 cm among children aged 6–59 months in Karamoja, Uganda. A secondary objective of the study is to determine the optimal WAZ and MUAC thresholds for detecting children with WaSt among children aged 6–59 months in Karamoja, Uganda.

## 2 METHOD

### 2.1 Study design and data source

This was a secondary data analysis of a database consisting of seven FSNA cross‐sectional survey datasets collected between June 2015 and July 2018 in all seven districts in Karamoja, Uganda. FSNA is usually conducted twice a year; in May/June for the lean (hunger or preharvest) and in November/December for the nonlean (postharvest) food security seasons. The FSNA is a population‐based cross‐sectional survey adapted from the Standardized Monitoring and Assessment of Relief and Transitions (SMART) survey protocol (Golden et al., 2006). The SMART methodology is a simplified and standardized household‐level survey methodology used to determine the public health situation in humanitarian and nonhumanitarian settings (Golden et al., 2006). The FSNA used a two‐stage cluster sampling approach. In the first stage, clusters were selected from an updated list of parishes in each district by using probability proportional to size sampling procedure. The second stage of sampling utilized systematic random sampling of households in each cluster. The target population for FSNA was children aged 0–59 months and their mothers or caregivers in the households. The sample size for FSNA was estimated based on the prevalence of global acute malnutrition and mortality rate for each district. About 20 to 30 households per cluster were sampled per district in each round of the survey (UNICEF, DFID, WFP, FAO, & IBFAN, 2018).

Trained field assistants collected data using a semistructured questionnaire administered with Open Data Kit software. The data collected included demographic and household characteristics. Anthropometric data collected included length/height, weight and MUAC among children 6–59 months. Age in months was estimated by maternal/career recall, with the aid of a local event calendar or extracted from child health record cards, when these were available (UNICEF, DFID, et al., 2018). FSNA surveys were conducted with support of the United Nations Children's Fund (UNICEF), the Food and Agriculture Organization (FAO) and the World Food Programme (WFP), in collaboration with the Department of Risk Reduction at the Office of the Prime Minister and the Ministry of Health, Uganda. The Makerere University School of Public Health and the International Baby Food Action Network (IBFAN), Uganda, were engaged to conduct the surveys.

### 2.2 Study setting

Karamoja is located in north‐eastern Uganda and borders Kenya and South Sudan. It has seven administrative districts: Moroto, Napak, Amudat, Nakapiripiriti, Kotido, Kaabong and Abim (Powell, 2010). Karamoja is a semi‐arid agro‐pastoral region with a history of decades of conflicts and cattle raiding (Powell, 2010). Food insecurity is persistently high; over half of its 1.4 million population are food insecure (Uganda IPC Technical Working Group, 2019). The region is highly dependent on food rations, provided mostly by United Nations agencies as well as other development partners. Children younger than 5 years in the Karamoja Region compared with children in other parts of Uganda have a higher risk of death. It is estimated that child mortality is 102 deaths per 1,000 live births in Karamoja, compared with 64 per 1,000 live births nationally (UBOS & ICF, 2018).

### 2.3 Study participants

Children aged 6–59 months with complete information on sex, MUAC, WAZ, WHZ and HAZ were included in this analysis. We identified and removed duplicate records from the database. Additionally, we removed children with bilateral pitting oedema and implausible anthropometric measurements such as height <45 and >120 cm and MUAC >20 cm (MoH Uganda & UNICEF, 2016). Implausible z‐scores were removed based on WHO's biological plausibility anthropometric criteria for z‐score outliers: HAZ <−6 and HAZ >6, WAZ <−6 and WAZ >5 and WHZ <−5 and WHZ >5 (WHO, 2009).

### 2.4 Case definitions

We defined wasted, stunted and underweight based on the z‐scores of the 2006 WHO growth standards (WHO, 2006) as follows:

Wasted: weight for height z‐score (WHZ) <−2.0.

Stunted: height for age z‐score (HAZ) <−2.0.

Underweight: weight for age z‐score (WAZ) <−2.0.

Severity of anthropometric deficits were defined as: no deficit ≥−2; moderate is <−2 and ≥−3 and severe <−3.

WaSt case was defined as concurrent WHZ<−2.0 and HAZ <−2.0 (Myatt et al., 2018).

Acute malnutrition was defined as wasting (WHZ<−2) and/or MUAC <12.5; severe acute malnutrition (SAM) as severe wasting (WHZ <−3) and/or MUAC <11.5 cm and moderate acute malnutrition (MAM) as moderate wasting (WHZ ≥−3 to −2) and/or MUAC ≥11.5 cm and ≤12.5 cm (UNHCR & WFP, 2011).

### 2.5 Data management and analysis

The names, types, length, coding schemes, units of measures of the variables and file format in each of the seven survey datasets were standardized and combined into one database (Myatt et al., 2018). Statistical analyses were conducted using STATA 13.0, SPSS Statistics 23 and Microsoft Excel. We analysed anthropometric z‐scores in STATA 13.0 (Leroy, 2011; WHO, 2009). A Venn diagram analysis was used to assess the intersection of children with WHZ <−2, HAZ <−2, WAZ <−2 and MUAC <12.5 cm (Venn, 1880). We adjusted for clustering effect of the multistage sampling during data analysis. We described the prevalence of WaSt and the characteristics of children with WaSt using summary statistics. We summarized continuous variables with medians and interquartile ranges (IQRs). We used percentages to describe categorical variables. We used chi‐square to test the significance of differences between the proportions of children with and without nutritional deficits. After testing for normality of the continuous variables, we used Wilcoxon rank‐sum test to compare the median values because they were not normally distributed (Bonita, Beaglehole, & Kjellström, 2006). Similar to an earlier analysis, we used Mann–Whitney *U* test to calculate the common language effect size (CLES) statistic to assess the probability of a higher median of WHZ and HAZ in wasted‐only, stunted‐only compared with WaSt cases (Conroy, 2012; Myatt et al., 2018). Receiver operating characteristic (ROC) curves and the Youden Index (sensitivity + specificity—100%) were used to determine optimal cut‐off values of MUAC and WAZ for detecting WaSt in SPSS Statistics 23 (Myatt et al., 2018; Youden, 1950).

### 2.6 Ethical considerations

We received approval to access the Karamoja FSNA datasets from the Office of the Prime Minister, Uganda. We sought a waiver of consent to use the FSNA datasets for this study from the Makerere University School of Medicine Higher Degrees Research and Ethics Committee. Ethical approval for the present analysis was granted by the Makerere University School of Medicine Higher Degrees Research and Ethics Committee and the Uganda National Council for Science and Technology.

## 3 RESULTS

### 3.1 Study participants profile

**Figure 1:**
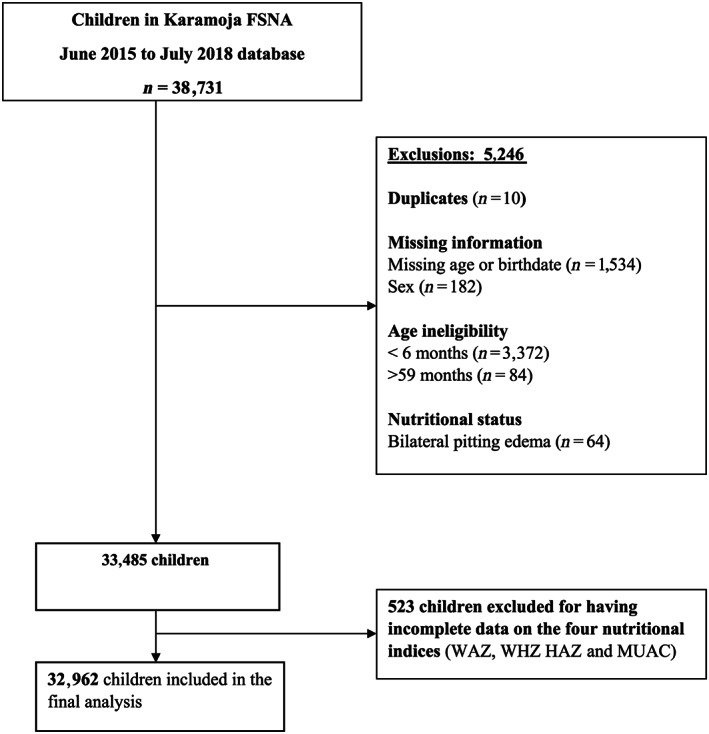
Flow chart showing participant selection among children 6–59 months in Karamoja; FSNA June 2015–July 2018 database

The final dataset contained data from 32,962 children aged 6–59 months. Although there was no sex difference (males: 49.67% vs. 48.95%; *p* = .745), the age distribution varied statistically (median: 26 vs. 19 months; *p* < .001) between the children in our study sample and those excluded for having incomplete anthropometric data. The flowchart (Figure 1) illustrates how eligible children were selected. The median age was 26 months (IQR: 15, 38) and 44.27% of the children were aged 6–23 months (Table 1).

**Table 1:** Characteristics of children aged 6–59 months in Karamoja; June 2015–July 2018 Food Security and Nutrition Assessment database

Attribute	Sample size (*N*)	Percent(%)
Children	32,962	100.00
Sex		
Male	16,372	49.67
Female	16,590	50.33
Age (months)		
Median (IQR^a^)	26 (15, 38)	
Age group (months)		
6–23	14,593	44.27
24–59	18,369	55.73
Survey period		
June 2015	5,023	15.24
December 2015	3,243	9.84
May 2016	4,623	14.03
December 2016	3,236	9.82
June 2017	5,942	18.03
January 2018	5,718	17.35
July 2018	5,177	15.71

a IQR, interquartile range.

### 3.2 Prevalence of undernutrition

Stunting was more frequent (33.58%) in the study sample than underweight (25.96%), wasting (12.03%) and low MUAC (10.58%; Table 2).

**Table 2:** Nutritional status of children in the sample, FSNA June 2015–July 2018 database

*N* = 32,962	WAZ	WHZ	HAZ	MUAC
Nutritional status^b^^,^ a	*n* b (%)	*n* (%)	*n* (%)	*n* (%)
No deficit	24,404 (74.04)	28,996 (87.97)	21,894 (66.42)	29.473 (89.42)
Moderate	6,099 (18.50)	3,027 (9.18)	6,839 (20.75)	2,811 (8.53)
Severe	2,459 (7.46)	9,39 (2.85)	4,229 (12.83)	6,78 (2.06)
Sum of moderate and severe	8,558 (25.96)	3,966 (12.03)	11,068 (33.58)	3,489 (10.58)

a WAZ, underweight; WHZ, wasted; HAZ, stunted; MUAC, low mid‐upper arm circumference.

b n, number of children/cases.

### 3.3 WaSt prevalence and overlap of underweight, stunting, wasting and low MUAC

**Figure 2:**
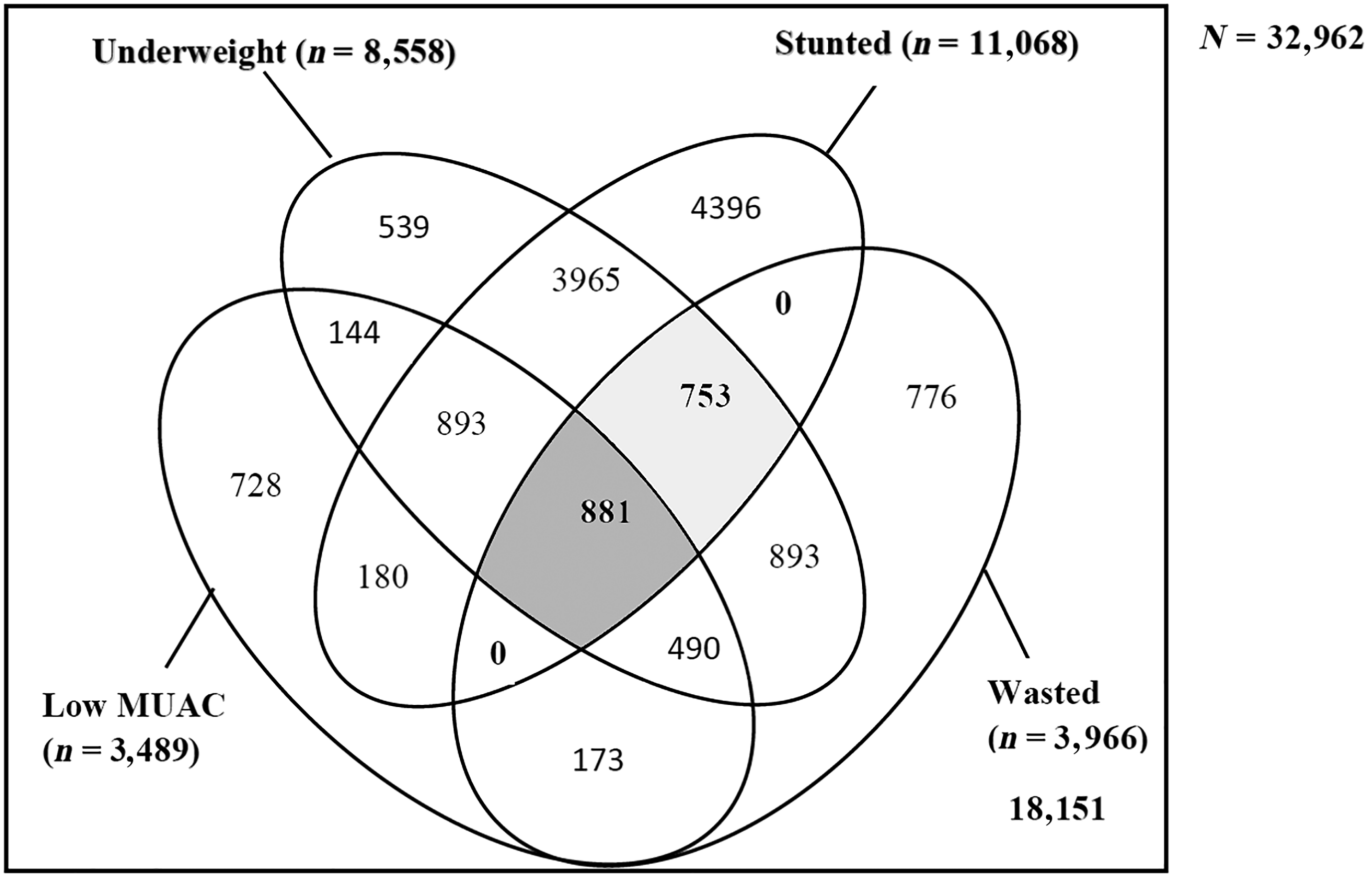
A Venn diagram showing the overlap of WHZ <−2.0, HAZ<−2.0, WAZ <−2.0 and MUAC <12.5 cm children. The two shaded areas depict WaSt in the study sample; the dark grey shaded portion depicts overlap of all the four anthropometric deficits

Nearly half (44.93%) of the children had at least one form of anthropometric deficit (Figure 2). The prevalence of WaSt children was 4.96% (95% CI [4.64, 5.29]), the two shaded portions of the Venn diagram show the intersection of sets of children with wasting and stunting (1,634/32,962). The dark grey shaded portion shows that approximately 2.67% (95% CI [2.45, 2.91]) of the sample (881/32,962) were currently wasted, stunted, underweight and had low MUAC. About half (53.92%; 881/1634) of the WaSt children had low MUAC, and all WaSt children were also underweight.

### 3.4 Wasting, stunting and WaSt by food security seasons and year of the survey

**Figure 3:**
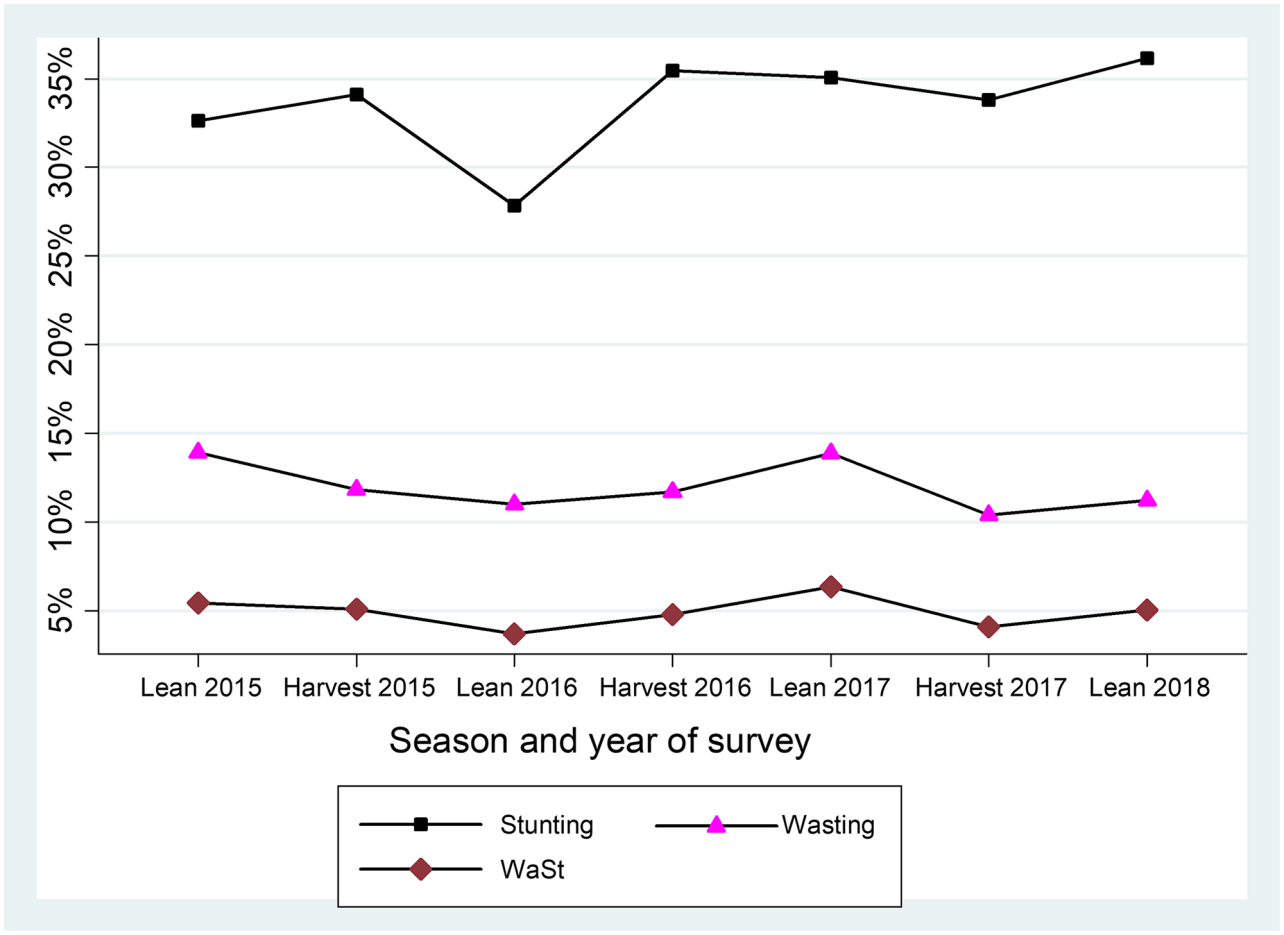
Trends of prevalence of wasting, stunting and children with WaSt children stratified by season and year of survey in Karamoja

The prevalence of wasting (12.57%) was higher in the lean season than in the harvest season (11.11%). The prevalence of stunting ranged from 31.00% in 2016 to 36.14% in 2018. There were more children with WaSt in the lean season than in the harvest season (5.21% vs. 4.53%; *p* = .0182; Table 3). With the exception of lean season in 2016, WaSt prevalence increased in all the lean seasons compared with the harvest seasons during the survey period (Figure 3).

**Table 3:** Prevalence of wasting, stunting and WaSt by season and year of the survey among children aged 6–59 months in Karamoja; FSNA June 2015–July 2018 database

Attribute	Sample size (*N*)	Wasted (*n* c, %)	Stunted (*n*, %)	WaSt (*n*, %)	95% CI^d^	*p* value^e^
Season of survey						
Lean^a^	20,765	2,611 (12.57)	6,882 (33.14)	1,081(5.21)	4.78–5.67	0.018
Harvest^b^	12,197	1,355 (11.11)	4,186 (34.32)	553 (4.53)	4.16–4.94	
Year of survey						
2015	8,266	1,082 (13.09)	2,745 (33.21)	437 (5.29)	4.67–5.98	0.016
2016	7,859	886 (11.27)	2,436 (31.00)	325 (4.14)	3.60–4.74	
2017	11,660	1,417 (12.15)	4,016 (34.44)	611 (5.24)	4.86–5.65	
2018	5,177	581 (11.22)	1871 (36.14)	261 (5.04)	4.33–5.87	

a Lean season stands for surveys conducted in May, June and July.

b Harvest season stands for surveys conducted in December and January.

c *n*, number of children/cases.

d CI, confidence intervals for proportion of WaSt.

e Proportions of WaSt were compared using chi‐square (χ^2^).

### 3.5 Underweight, low MUAC, stunted, wasted and WaSt children by age groups

**Figure 4:**
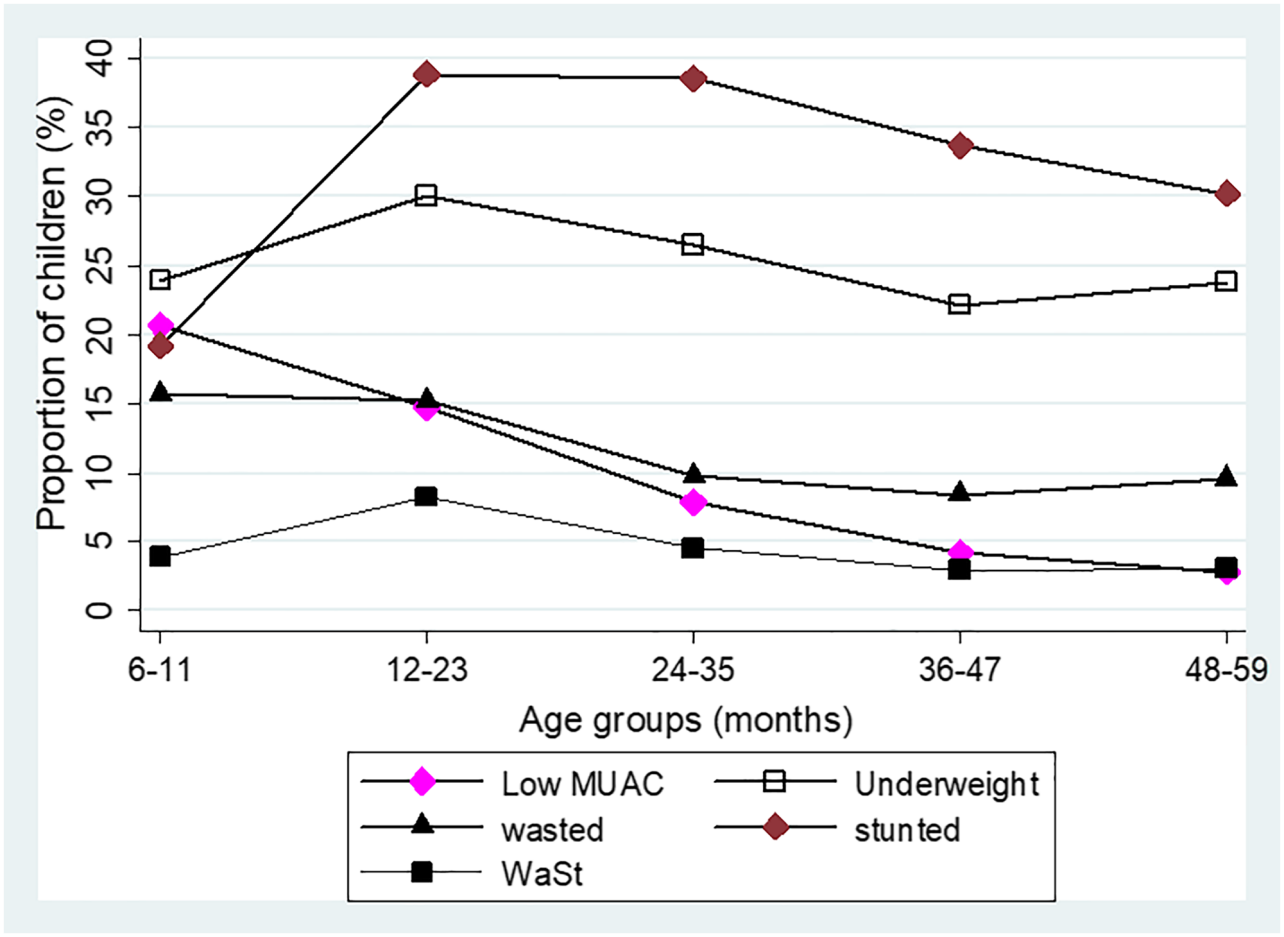
Proportions of underweight, low MUAC, stunted, wasted and WaSt children stratified by age groups in Karamoja

Underweight (23.96%) followed by low MUAC (20.72%) were the most prevalent forms of undernutrition in children aged 6–11 months (Figure 4). Wasting was prevalent at age 6–11 months (15.74%) and 11–23 months (15.27%) but declined among children >24 months. The proportion of children with stunting, underweight and WaSt peaked at age 12–23 months, whereas the proportion of children with low MUAC reduced from 14.79% at 12–23 months to 2.77% at 49–59 months. Stunting was markedly prevalent between the ages of 12–35 months. The prevalence of underweight and WaSt had a parallel pattern, and both conditions were more frequent in children aged 6–35 months (Figure 4).

### 3.6 WaSt and low MUAC by age groups and sex

**Figure 5:**
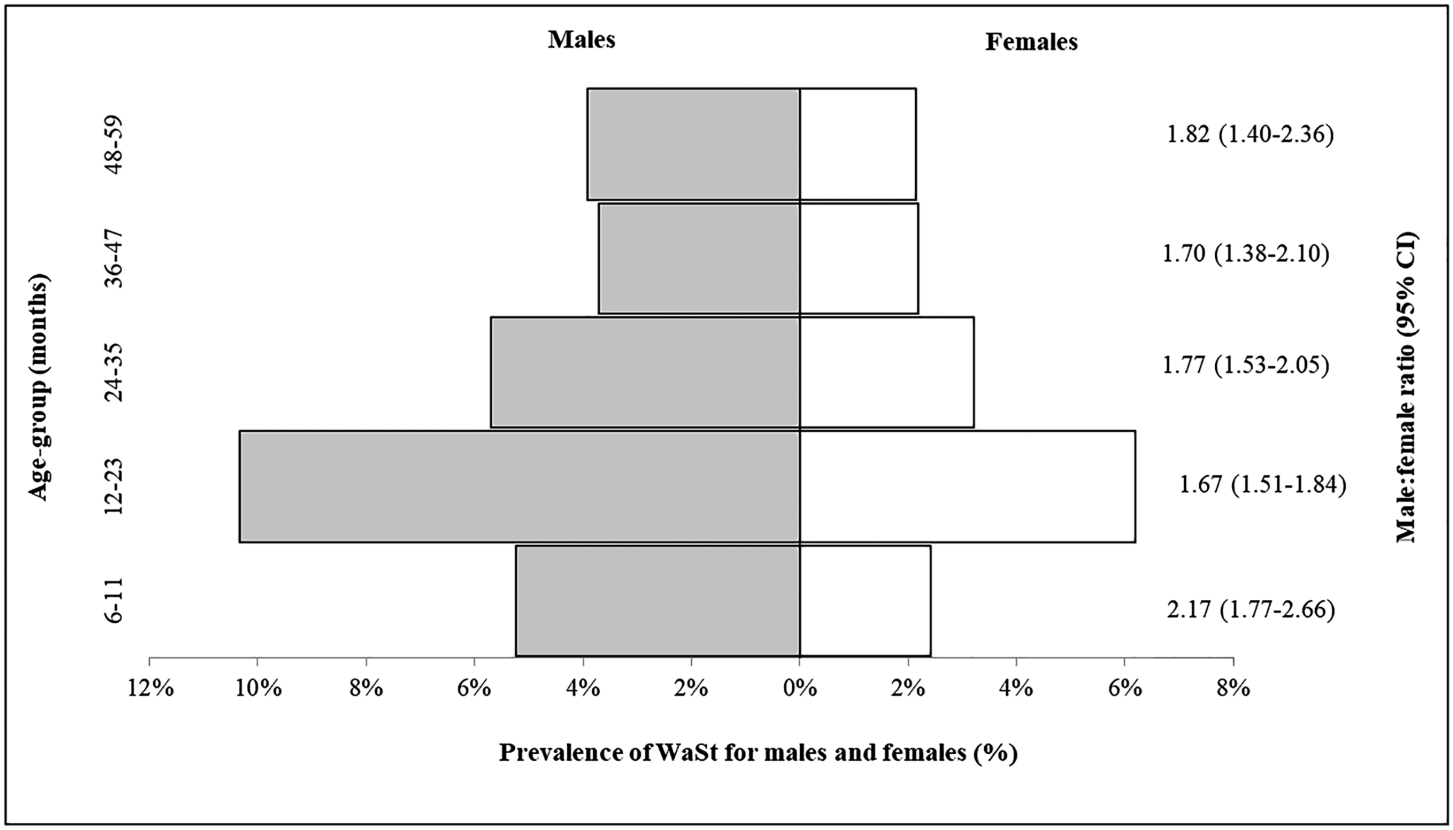
Male to female prevalence ratio of WaSt by age group among children 6–59 months in Karamoja. Note: Male:female ratio presented with 95% confidence interval (95% CI). The grey shaded area of the graph shows prevalence of WaSt among males versus the unshaded area for prevalence of WaSt among females in each age group

Compared with females, males had a higher prevalence of wasting (14.16% vs. 9.93%) and stunting (37.00% vs. 30.25%). WaSt was also more frequent in males (6.34%; 95% CI [5.91, 6.80]) than in females (3.59%; 95% CI [3.24, 3.98]). The overall male to female WaSt prevalence ratio was 1.77 (95% CI [1.60, 1.95]). WaSt prevalence peaked at 12–23 months in both sexes. Among children aged 12–23 months, WaSt was more prevalent in males than females (Figure 5). WaSt prevalence was consistently higher in males in all age groups. In a subpopulation analysis of children with WaSt (*n* = 1,634), there were more WaSt‐only cases (51.25%; 95% CI [48.05, 54.44]) compared with those who had both WaSt and low MUAC (48.75%; 95% CI [45.56, 51.95]) among the males (Table 4). More females had WaSt and low MUAC (62.92%; 95% CI [58.91, 66.75]) than those with WaSt‐only (37.08%; 95% CI [33.25, 41.09]). The difference between the median MUAC in males with WaSt compared with females with WaSt was significant (12.50 vs. 12.10 cm; *p* < .001). The median MUAC among children with WaSt and low MUAC was slightly higher in males than in females (11.90 vs. 11.70 cm; *p* = .0001; Table 4).

**Table 4:** Sex and age of children with WaSt and low MUAC versus WaSt‐only; and MUAC measurement among males versus females among WaSt children aged 6–59 months in Karamoja; FSNA June 2015–July 2018 database

Attribute	WaSt and low MUAC (*n* b=881)	WaSt‐only (*n* = 753)	*p* value^a^
Sex			
Males	48.75 (45.56, 51.95)	51.25 (48.05,54.44)	<.001
Females	62.92 (58.91, 66.75)	37.08 (33.25, 41.09)	
Age group			
6–23 months			
Males	58.86 (54.35, 63.23)	41.14 (36.77, 45.65)	.0001
Females	75.50 (69.46, 80.67)	24.50 (19.33, 30.54)	
24–59 months			
Males	34.04 (30.33, 37.96)	65.96 (62.04, 69.67)	.003
Females	44.90 (38.71, 51.24)	55.10 (48.76, 61.29)	
MUAC measurement (median [IQR])^c^ by sex			
Attribute	Males (*n* = 1,038)	Females (*n* = 596)	
All WaSt	12.50 (11.90, 13.00)	12.10 (11.50, 12.70)	<.001
WaSt and low MUAC	11.90 (11.40, 12.10)	11.70 (11.20, 12.00)	.0001
WaSt‐only	13.00 (12.70, 13.50)	12.90 (12.70, 13.40)	.231

a Proportions were compared using chi‐square (χ^2^), and continuous variables were compared using Wilcoxon rank‐sum test.

b IQR, interquartile range.

c *n*, number of children/cases.

### 3.7 The degree of anthropometric deficit among children with WaSt compared with wasted‐only and stunted‐only

There was a similar median WHZ in children with wasting only and those with WaSt (−2.49 vs. −2.51; *p* = .123) with probability of superiority of 51.43% (95% CI [49.61, 53.26]). The CLES showed a slightly superiority WHZ (wasted‐only) > WHZ (WaSt), although the difference was not statistically significant. The median HAZ in stunted‐only children compared with WaSt children was significantly different (−2.74 vs. −2.97; *p* < .001). The probability of HAZ in a random stunted‐only case being above HAZ in a WaSt case was 58.07% (95% CI [56.56, 59.58]). The CLES is >50% and the 95% CI does not include 50% that showed a clear ‘superiority’ of HAZ (stunted‐only) > HAZ (WaSt). WaSt children also had a significantly lower median WAZ than children with wasting only (−3.48 vs. −2.17; *p* < .001) or stunting only (−3.48 vs. −2.03; *p* < .001). Children with WaSt had a lower median MUAC compared with children with wasting only (12.40 vs. 13.00; *p* < .001) and stunting only (12.40 vs. 13.70; *p* < .001; Table 5).

**Table 5:** Comparison of anthropometric characteristics and age of WaSt versus wasted‐only and WaSt versus stunted‐only children aged 6–59 months in Karamoja; FSNA June 2015–July 2018 database

Attribute	All children (*N* = 32,962)	WaSt (*n* c=1,634)	Wasted‐only (*n* = 2,332)	*p* value^a^	Stunted‐only (*n* = 9,434)	*p* value^b^	Probability of superiority (95% CI)^e^
dZ‐scores							
WHZ	−0.72 (−4.94, 0.01)	−2.51 (−2.98, −2.23)	−2.49 (−2.95, −2.21)	.123			51.43 (49.61–53.26).
HAZ	−1.43 (−2.35, −0.46)	−2.97(−3.65,−2.97)			−2.74 (−3.32, −2.33)	<.001	58.07 (56.56–59.58)
WAZ	−1.24 (−2.04, −0.48)	−3.48 (−3.91–3.08)	−2.17 (−2.57, −1.68)	<.001	−2.03 (−2.53, −1.48)	<.001	
Anthropometric measurements^d^							
Weight	10.40 (8.40, 12.70)	7.40 (6.60, 8.60)	8.60 (7.10, 11.10)	<.001	9.90 (8.40, 11.50)	<.001	
Height	82.20 (74.00, 91.70)	74.30 (69.90, 80.00)	81.30 (72.30, 93.95)	<.001	79.50 (73.30, 85.80)	<.001	
MUAC	13.90 (13.10, 14.80)	12.40 (11.70, 13.00)	13.00 (12.40, 13.60)	<.001	13.70 (13.00, 14.50)	<.001	
dAge (months)							
	26.00 (15.00, 38.00)	20.00 (14.13–30.00)	19.00 (11.00, 36.00)	.0028	27.17 (18.14, 38.0)	<.001	

a Median values of WaSt and wasted‐only were compared using Wilcoxon rank‐sum test.

b Median values of WaSt and stunted‐only were compared using Wilcoxon rank‐sum test.

c *n*, number of children/cases.

d Median (IQR) interquartile range.

e Probability superiority or common language effect size statistic (CLES) with confidence intervals (CI) derived based on Mann–Whitney statistics.

### 3.8 WaSt case detection using WAZ and MUAC

The WAZ <−2.60 cut‐off point detected WaSt with an excellent sensitivity (99.02%) and a high specificity (90.71%) with an area under the receiver operator characteristic curve (AUC) of 0.980 (95% CI [0.978, 0.981]; Table 6). The optimal MUAC cut‐off of 13.20 cm detected WaSt cases with a good sensitivity of 81.58% and a moderate specificity at 76.15%. The area under the ROC curve for MUAC <13.20 cm cut‐off was 0.863 (95% CI [0.855, 0.872]; Table 6).

**Table 6:** MUAC and WAZ cut‐offs for detecting WaSt cases among children 6–59 months in Karamoja; FSNA June 2015–July 2018 database

Anthropometric indicators	Sensitivity (%)	Specificity (%)	Youden index (%)	AUC^a^ (95% CI^b^)
WAZ −2.55 (−2.60)^c^	99.02 (98.49–99.55)	90.71 (90.39–91.03)	89.73	0.980 (0.978–0.981)
MUAC <13.15 cm (13.20 cm)^c^	81.58 (79.70–83.46)	76.15 (75.78–76.62)	57.73	0.863 (0.855–0.872)

a AUC, area under the receiver operator characteristic curve.

b CI, confidence interval.

c Rounded up WAZ <−2.55 to WAZ <−2.60 and 13.15 to 13.20 cm for practical purposes because these indicators are measured to one decimal.

## 4 DISCUSSION

We found a pooled WaSt prevalence of 4.96% in our analysis. Previous studies reported WaSt prevalence between 1% and 3% (Khara et al., 2018; Saaka & Galaa, 2016). The variation in WaSt prevalence seen in our study compared with previous studies could be due to differences in age group of children, food insecurity, agro‐pastoral practices, ethnicity and social stability.

It was not surprising that about 5% of the children in our analysis had WaSt considering the high wasting and stunting prevalence in Karamoja. Nevertheless, the WaSt prevalence observed has public health implications. First, some children with WaSt risk being missed or misclassified as moderately wasted in the current therapeutic feeding programme that only focuses on SAM and MAM (MoH Uganda & UNICEF, 2016). Further, risk of death among WaSt children is similar to severely wasted children (McDonald et al., 2013; Myatt et al., 2018; Odei Obeng‐Amoako et al., 2020). Therefore, detecting and treating WaSt children within the existing nutrition and child survival interventions could substantially reduce child mortality (Garenne et al., 2019; Myatt et al., 2018; Myatt et al., 2019). There is an evidence gap in WaSt prevalence in high undernutrition burden settings. Routine national and subnational level nutrition surveys such as FSNA and SMART surveys need to be modified to include WaSt indicators to inform programme and policy decision‐making.

WaSt prevalence varied by food security seasons in our study, similar to a Gambian study where being born at the start of the hunger season was a risk factor for early linear growth faltering and stunting (Schoenbuchner et al., 2019). These findings imply that WaSt prevalence could be seasonal and routine monitoring of WaSt prevalence would be required to inform effective detection and treatment.

About half (53.92%) of the children with WaSt had low MUAC. An overlap between WaSt and low MUAC implies a child had simultaneously experienced the four common anthropometric deficits: wasting, stunting, underweight and low MUAC. Also, all WaSt children were underweight, as previously reported (Myatt et al., 2018). Thus, WaSt could be a useful indicator of concurrent anthropometric deficits (i.e., wasting, stunting and underweight) for communicating programmatic and policy outcomes (Myatt et al., 2018). Further research is needed to validate this finding.

In the present analysis, the proportion of children with different forms of anthropometric deficits were associated with age. Both underweight and low MUAC were common among children aged 6–11 months. However, the proportion of children with low MUAC declined thereafter, underweight rate was higher at age 12–23 months but decreased steadily after 24 months. Wasting was common among children <24 months but less among >24 months old. Stunting was most prevalent beyond age 24 months. The higher underweight rate in the age group 36–48 months may indicate higher stunting rather than wasting rate (Waterlow, 1974). A child's body prioritizes weight gain over linear growth during nutritional recovery (Dewey et al., 2005). Wasting and stunting tend to be risk factors for each other (Myatt et al., 2018). A longitudinal study showed that wasting predicted the odds of stunting by three times. Conversely, stunting predicted the odds of wasting by two times (Schoenbuchner et al., 2019). Because of coexistence of growth faltering, integrated programming to address undernutrition in young children is needed (Bergeron & Castleman, 2012; Wells et al., 2019). Also, therapeutic feeding programmes should be optimized to include WaSt children and to promote linear growth in wasted children during treatment (Bergeron & Castleman, 2012; Khara & Dolan, 2014).

Younger children aged <36 months particularly males were more likely to have WaSt. The estimated WaSt male:female prevalence ratio is similar to findings reported in an earlier study (Myatt et al., 2018). Garenne et al. also showed that before age 30 months, males were 1.6 times more likely to be WaSt than females; however, the sex difference disappeared after age 30 months (Garenne et al., 2019). These findings indicate that male children aged <36 months have a higher risk of WaSt (Garenne et al., 2019; Khara et al., 2018; Myatt et al., 2018). Given that children under 5 years are at risk of undernutrition, all children especially those <36 months old irrespective of sex should be targeted through a community‐based WaSt screening programme.

Currently, the reasons for the observed sex difference in WaSt prevalence remain unclear and require further investigations. In a subpopulation analysis, we found that fewer males were WaSt and had low MUAC compared with those with WaSt‐only, whereas more females had WaSt and low MUAC than those with WaSt‐only. The sex disparities in childhood undernutrition could be due to the sex differences in body composition mainly muscle mass and body fat distribution measured by the different anthropometric indicators (Admassu et al., 2018; Park, Park, Kim, Kim, & Chung, 2011). Future studies should analyse the overlaps between wasting, stunting, underweight and MUAC to clarify understanding of sex and age patterns of WaSt.

In the present analysis, children with WaSt were more severely stunted than stunted‐only children. The level of wasting did not differ between wasted‐only and WaSt cases. This is contrary to previous studies where WaSt cases were both severely wasted and stunted compared with wasted‐only or stunted‐only cases (Garenne et al., 2019; Myatt et al., 2018). The reason for worse stunting among the WaSt cases could be indicative of chronic hunger given that Karamoja is perennially food insecure (Uganda IPC Technical Working Group, 2019). Therefore, prompt WaSt case detection can improve treatment coverage and increase child survival in settings with high malnutrition burden.

Thresholds of WAZ <−2.60 and MUAC <13.20 cm were useful for detecting WaSt cases with optimum performance. WAZ <−2.60 cut‐off detected WaSt cases at an excellent sensitivity and a high specificity. These WAZ and MUAC cut‐offs were similar to the thresholds found in an earlier study (Myatt et al., 2018). MUAC <13.20 cm performed optimally to detect WaSt with good sensitivity but with a lower specificity. WAZ <−2.60 identified additional WaSt cases that MUAC did not identify. Therefore, WAZ had a better performance than MUAC in detecting WaSt cases. We rounded up WAZ <−2.55 to WAZ <−2.60 and 13.15 to 13.20 cm to make the cut‐offs user friendly because these indicators are measured to one decimal, although the cut‐offs could slightly increase the number of WaSt cases.

Few published data on anthropometric indicators for screening and detecting WaSt exist. A recent study reported that WAZ <−2.60 and MUAC <13.30 cm performed considerably better than chance to identify WaSt cases with the areas under the ROC curves of 0.9726 for WAZ and 0.8759 for MUAC (Myatt et al., 2018). In another analysis, Myatt et al. found that a combination of WAZ <−2.80 and MUAC ≥11.50 cm effectively identified WaSt children at risk of death (Myatt et al., 2019). Currently, WAZ is the routine indicator for growth faltering in growth monitoring and promotion (GMP) and integrated management of childhood illnesses (IMCI) at the health facilities (Ashworth, Shrimpton, & Jamil, 2008). MUAC is an independent admission criterion for therapeutic feeding programmes (WHO, 2013). Integration of MUAC into GMP and IMCI and WAZ in therapeutic feeding programmes should be considered to facilitate WaSt case detection and to expand treatment coverage in settings with high undernutrition burden. However, further research on the appropriateness of WAZ and MUAC cut‐off points identified in this study to detect WaSt cases, monitor treatment responses and post‐treatment relapse would be required to inform future decision‐making on wasting and WaSt.

### 4.1 Strengths and limitations

The dataset used in this analysis was collected based on a standardized protocol during each round of the survey. However, our study is not devoid of limitations usually associated with retrospective analysis. Random error is a common challenge associated with a fixed sample size of a historic dataset. Nonetheless, random error associated with secondary dataset was likely minimized because of the large sample size of the dataset and the probability proportional to population size sampling procedure used in the FSNA surveys (Hulley et al., 2001). Selection bias is an inherent limitation of existing datasets. We cleaned and censored incomplete data, missing data and implausible anthropometric data to minimize selection bias. Children aged 6–59 months who had complete anthropometric data were eligible for the present analysis. There was no sex difference between children included in the present analysis and those who were excluded due to incomplete anthropometric data. However, children in this study were older than those excluded for having incomplete data on the four anthropometric indicators. Our findings should be interpreted in light of this potential selection bias. Information bias is another limitation associated with a secondary dataset. The sources of information bias could be due to missing data and erroneous anthropometric measurements by field enumerators. Regular and standardized training of field enumerators, the use of standard operating procedures for sampling and collecting data in FSNA surveys probably minimized information bias in datasets. Causal inference about WaSt could not be made in our analysis because the design was cross‐sectional. However, the findings could be useful baseline information for future studies on WaSt prevalence and case detection. The evidence on WaSt prevalence estimates and case detection would be useful for decision‐making on WaSt programming and planning.

## 5 CONCLUSION

WaSt prevalence of 5% found in Karamoja is a public health concern given the increased risk of death associated with WaSt. All four anthropometric deficits, underweight, wasting, stunting and low MUAC, occurred simultaneously in half of the WaSt cases. Some of the WaSt cases did not have low MUAC. Our analysis suggests that WAZ and MUAC could be useful tools to detect WaSt cases with optimal performance. However, future prospective studies on WAZ and MUAC cut‐offs for WaSt detection in different populations would be required. Routine prevalence reports are useful for decision‐making. Future consideration to include MUAC into GMP and IMCI and WAZ into therapeutic feeding programmes to detect WaSt for treatment is recommended.

## CONFLICTS OF INTEREST

The authors declare that they have no conflicts of interest.

## CONTRIBUTIONS

GAOOA, HW, MM, AB, JC, BKM, JNK and CAK conceptualized and designed the research study. GAOOA, HW, RA, JC, JNK, MM, AB and CAK analysed and interpreted the data. GAOOA, HW, JNK and CAK drafted the initial manuscript. GAOOA, HW, BKM, JNK, EM, IO, ALO, RA, JC, MM, AB and CAK reviewed and finalized manuscript.
